# Biomarkers of Airway Disease, Barrett’s and Underdiagnosed Reflux Noninvasively (BAD-BURN) in World Trade Center exposed firefighters: a case–control observational study protocol

**DOI:** 10.1186/s12876-024-03294-9

**Published:** 2024-08-09

**Authors:** Urooj Javed, Sanjiti Podury, Sophia Kwon, Mengling Liu, Daniel H. Kim, Aida Fallahzadeh, Yiwei Li, Abraham R. Khan, Fritz Francois, Theresa Schwartz, Rachel Zeig-Owens, Gabriele Grunig, Arul Veerappan, Joanna Zhou, George Crowley, David J. Prezant, Anna Nolan

**Affiliations:** 1 Department of Medicine, Division of Pulmonary, Critical Care and Sleep Medicine, New York University Grossman School of Medicine (NYUGSoM), New Bellevue, 16 North Room 20 (Lab), 462 1st Avenue, New York, NY 10016 USA; 2 Department of Population Health, Division of Biostatistics, NYUGSoM, New York, NY USA; 3 Center for Esophageal Health, NYUGSoM, New York, NY 10016 USA; 4 Department of Medicine, Division of Gastroenterology, NYUGSoM, New York, NY 10016 USA; 5 Fire Department of New York, Bureau of Health Services, Brooklyn, NY 1120 USA; 6 Department of Medicine, Division of Environmental Medicine, NYUGSoM, New York, NY 10010 USA

**Keywords:** Air pollutants, Airway hyperreactivity, Ambient particulate matter, Barrett’s esophagus, Gastro-esophageal reflux disease, Particulate, Aerodigestive

## Abstract

**Background:**

Particulate matter exposure (PM) is a cause of aerodigestive disease globally. The destruction of the World Trade Center (WTC) exposed first responders and inhabitants of New York City to WTC-PM and caused obstructive airways disease (OAD), gastroesophageal reflux disease (GERD) and Barrett’s Esophagus (BE). GERD not only diminishes health-related quality of life but also gives rise to complications that extend beyond the scope of BE. GERD can incite or exacerbate allergies, sinusitis, bronchitis, and asthma. Disease features of the aerodigestive axis can overlap, often necessitating more invasive diagnostic testing and treatment modalities. This presents a need to develop novel non-invasive biomarkers of GERD, BE, airway hyperreactivity (AHR), treatment efficacy, and severity of symptoms.

**Methods:**

Our observational case-cohort study will leverage the longitudinally phenotyped Fire Department of New York (FDNY)-WTC exposed cohort to identify *Biomarkers of Airway Disease, Barrett’s and Underdiagnosed Reflux Noninvasively (BAD-BURN)*. Our study population consists of *n =* 4,192 individuals from which we have randomly selected a sub-cohort control group (*n =* 837). We will then recruit subgroups of *i.* AHR only *ii.* GERD only *iii.* BE *iv.* GERD/BE and AHR overlap or *v.* No GERD or AHR, from the sub-cohort control group. We will then phenotype and examine non-invasive biomarkers of these subgroups to identify under-diagnosis and/or treatment efficacy. The findings may further contribute to the development of future biologically plausible therapies, ultimately enhance patient care and quality of life.

**Discussion:**

Although many studies have suggested interdependence between airway and digestive diseases, the causative factors and specific mechanisms remain unclear. The detection of the disease is further complicated by the invasiveness of conventional GERD diagnosis procedures and the limited availability of disease-specific biomarkers. The management of reflux is important, as it directly increases risk of cancer and negatively impacts quality of life. Therefore, it is vital to develop novel noninvasive disease markers that can effectively phenotype, facilitate early diagnosis of premalignant disease and identify potential therapeutic targets to improve patient care.

**Trial registration:**

Name of Primary Registry: “Biomarkers of Airway Disease, Barrett's and Underdiagnosed Reflux Noninvasively (BADBURN)”. Trial Identifying Number: NCT05216133. Date of Registration: January 31, 2022.

**Supplementary Information:**

The online version contains supplementary material available at 10.1186/s12876-024-03294-9.

## Background

Particulate matter (PM) exposure is a risk factor for aerodigestive disease and mortality [1–3]. On September 11, 2001 (9/11), first-responders and inhabitants of New York City were exposed to World Trade Center (WTC)-PM [4–35]. Many subsequently developed aerodigestive diseases including obstructive airways disease (OAD), gastroesophageal reflux disease (GERD) and Barrett’s Esophagus (BE) [23, 34, 36–42]. By 2005, approximately 44% of WTC rescue and recovery workers had developed GERD, which is 8.2-fold higher than the pre-9/11 prevalence, and more than double the general US population [43–46]. After WTC-PM exposure, GERD occurred more often in asthmatics [42]. Comorbid aerodigestive disease affected 51.4% of firefighters [47].

GERD and BE are risk factors for esophageal adenocarcinomas (EAC) [48]. Patients with BE face at least 30-fold higher risk of developing EAC than the general population [49, 50]. Complications of GERD extend beyond malignancy and can adversely affect quality of life (QoL), impair productivity, and lifespan [46, 51–53]. GERD can incite or exacerbate co-morbidities such as allergies, sinusitis, chronic bronchitis, and asthma [54]. There is a 59.2% prevalence of GERD symptoms in patients with asthma compared to 38.1% in controls [55]. GERD treatment in WTC responders with proton pump inhibitors (PPIs) have been found to increase risk of severe cognitive impairment [56]. Cognitive decline with PPI use has also been reported in the general population [57].

Despite numerous studies suggesting potential interdependence between airway and digestive diseases, the underlying causative factors and mechanisms remain unclear [55]. Biomarkers are often key to identifying causative pathways and mechanistic targets. While some studies have investigated serum, salivary, and microbial biomarkers of GERD, they are often not focused on the contribution of respiratory disease [58–60].

The availability of clinical longitudinal phenotyping makes the WTC-PM exposed Fire Department of New York (FDNY) first responders cohort ideal for biomarker discovery [10, 22, 28–31, 61–65]. Notably, we have successfully identified biomarkers associated with GERD and BE in a pilot population with respiratory disease, facilitating the identification of biologically relevant immune pathways [3].

The diagnosis of GERD itself is a complex process that relies on subjective clinical symptoms and often necessitate objective but invasive testing such as endoscopy and 24-h pH monitoring [66]. Those with endoscopic evidence of reflux may be entirely asymptomatic, potentially leading to under-diagnosis of patients at risk of BE and EAC [67, 68]. Even with the most invasive procedures, the diagnosis of GERD can be elusive and plagued by poor sensitivity [69].

**Fig. 1:**
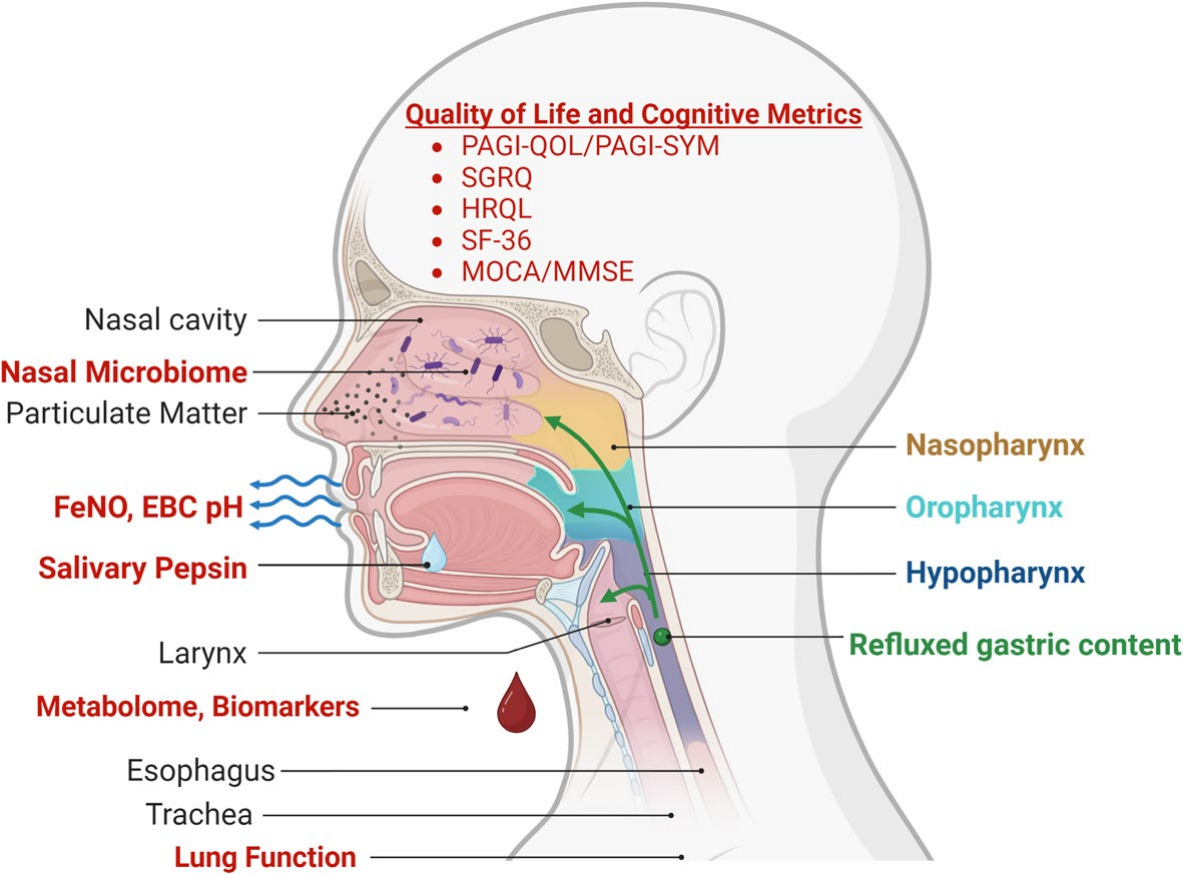
Overview of planned biomarker assessments

In light of this, we propose to explore noninvasive biomarkers that could identify a population of aerodigestive disease, enabling better phenotyping of FDNY-WTC cohort with aerodigestive disease. In addition to their diagnostic utility, noninvasive biomarkers may direct future research into mechanisms and their downstream effects. GERD/BE biomarkers are also important to identify in the clinically silent presentations [69]. Additionally, we will identify novel non-invasive biomarkers of aerodigestive disease through a multi-OMIC approach. We will profile not only the metabolome and microbiome, but also exhaled, secreted, and blood biomarkers of aerodigestive disease Fig. 1 [70].

To address a critical gap in the current literature, we will 1. *Quantify noninvasive measures* of aerodigestive disease (salivary pepsin, serum biomarkers/metabolome, fractional exhaled nitric oxide (FeNO), exhaled breath condensate (EBC), microbiome, cognitive measures and aerodigestive QoL/disease severity measures to phenotype and assess treatment efficacy. 2. *Develop and optimize* a noninvasive biomarker model of aerodigestive disease and also 3. Determine the effect of aerodigestive disease on QoL, cognition and symptom phenotype.

## Methods/design

### Study design and participants

The FDNY WTC-health program (WTC-HP) electronic medical record (EMR) will be used to obtain clinical variables such as age, gender, years of FDNY service, WTC site exposure level, and lung function measures, as previously described [22, 27, 62–65, 71]. Our observational study is NYU IRB Approved # 21–00679 and available at clinicaltrials.gov #NCT05216133. Study Definitions and Inclusion/Exclusion Criteria can be found in Table 1.

**Table 1:**
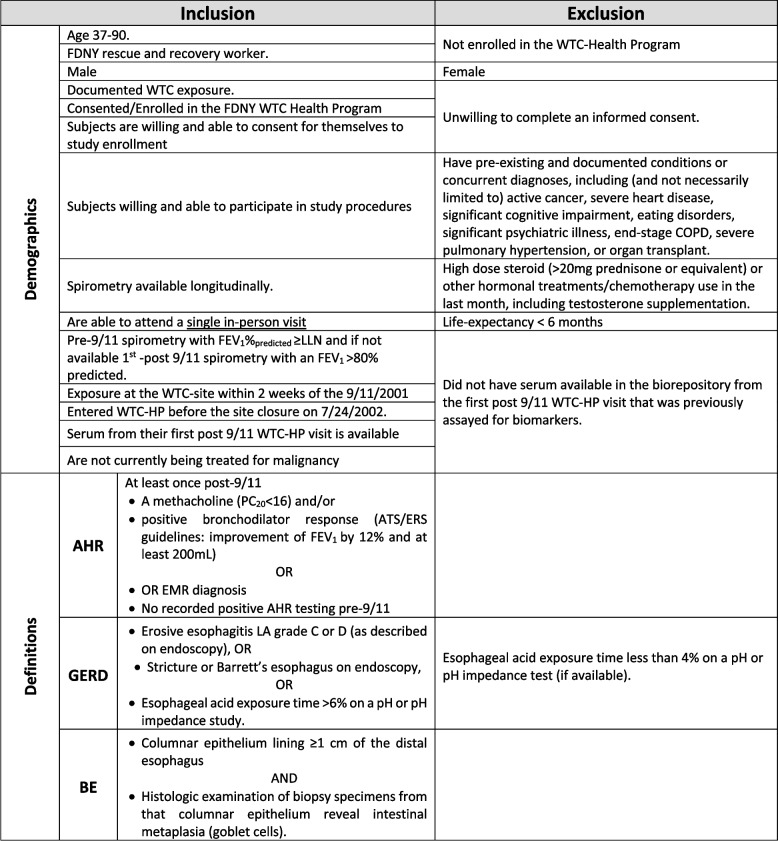
Inclusion and exclusion criteria

*FDNY* New York City Fire Department, *WTC* World Trade Center, *COPD* Chronic Obstructive Pulmonary disease, *FEV1* Forced expiratory volume in the first second, *LLN* lower limit of normal, *WTC-HP* World Trade Center Health Program, *PC20* provocative concentration of Methacholine, *AHR* airway hyperresponsiveness, *ATS/ERS* American Thoracic Society/European Respiratory Society, *EMR* Electronic Medical Record, *GERD* Gastroesophageal Reflux Disease, *BE* Barrett’s Esophagus

### Study oversight

It will be the responsibility of the principal investigator to oversee the safety of the study at his/her site. This safety monitoring will include careful assessment and appropriate reporting of adverse events, as well as the construction and implementation of a site data and safety-monitoring plan (Study Auditing, Monitoring and Inspecting). Medical monitoring will include a regular assessment of the number and type of adverse events. All modifications will be communicated to the IRB and will be reviewed.

### Data safety monitoring

The principal investigator will be responsible for overall data safety monitoring. The following data points will be monitored: Adverse events (AE) will be monitored. Data safety monitoring reviews will be conducted yearly to ensure the safety of subjects. There are no predefined halting rules in place. We do not foresee temporary suspension of enrollment and/or study intervention due to the intent to treat nature of the study intervention. Data Monitoring Committee is not needed due to minimal risk study.

### Study population

#### Source cohort

**Fig. 2:**
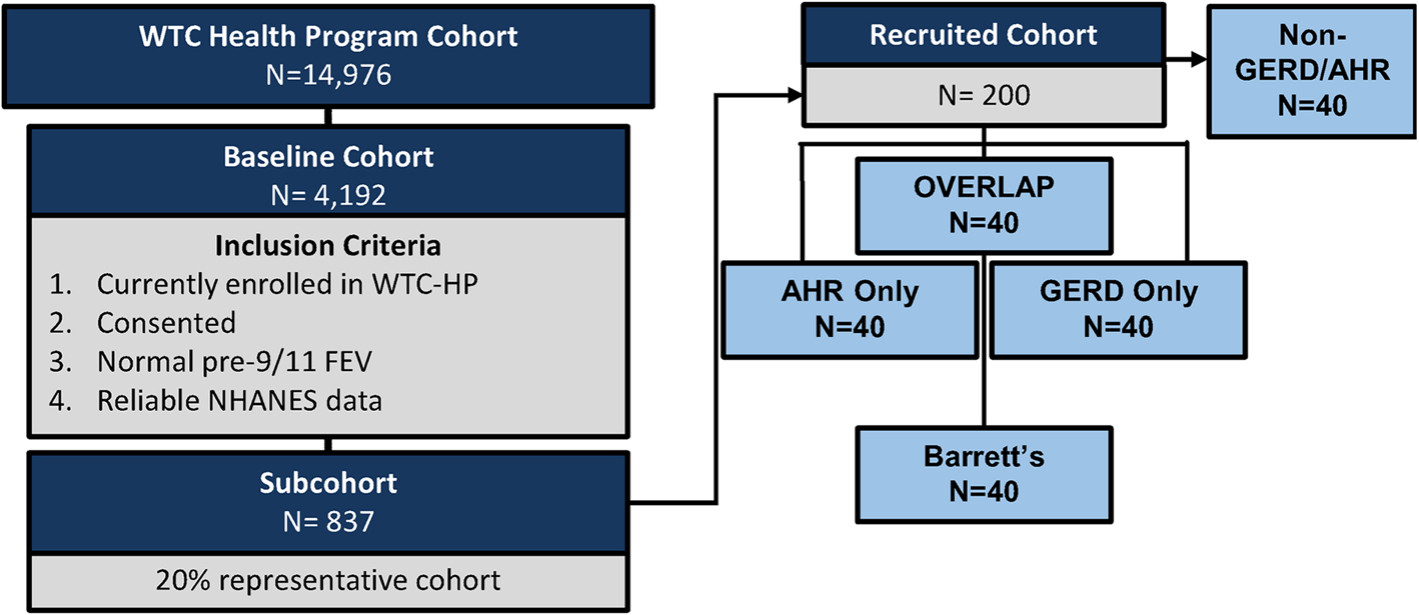
Study design

All participants in the WTC-HP (*n =* 14,976) were screened, Fig. 2. *Inclusion Criteria:* i. Actively consented and enrolled member of the WTC-HP. ii. Pre-9/11 spirometry with Forced Expiratory Volume in 1 s (FEV_1_) ≥ Lower Limit of Normal (LLN) iii. Male Firefighter status on 9/11 with exposure at the WTC-site and entry into WTC-HP before the site closure on 7/24/2002. *Exclusion Criteria:* i. lung disease prior to 9/11 as defined by positive methacholine or bronchodilator test, or FEV_1_ < LLN. ii. Not part of initial cohort in data extraction from August 1, 2017 [72]. After all inclusion/exclusion criteria applied, the baseline cohort consists of *n =* 4,192. *Sub-cohort Development.* A representative cohort of 20% was randomly selected (*n =* 837; SPSS v. 28) from the above baseline cohort, Fig. 2.

*Recruited Cohort* will be developed to assess for noninvasive biomarkers. We will recruit a subset *N =* 40/group (*i.* AHR only *ii.* GERD only *iii.* BE *iv.* GERD/BE and AHR overlap or *v.* No GERD nor AHR) from the sub-cohort, Fig. 2. Recruitment strategies will include: i. Direct mailings; ii. Email (potential participants will be sent the same IRB-approved recruitment message to their personal emails using end-to-end encryption; iii. Study website will include recruitment messages providing general information on the study and answers to frequently-asked questions. No direct communications will be made with participants through the website, and no PHI will be used or available within the study website; iv. Telephone contact. A description of the study will be provided to potential participants and, upon their expression of interest, the investigator will perform an eligibility screening. In addition to meeting the inclusion criteria as outlined above, participants should: i. have available serum from their first post 9/11 WTC-HP ii. Not currently be receiving treatment for malignancy iii. Have no limitations to a minimal risk blood draw iv. Be willing and able to sign consent; and v. be able to attend a single-visit.

All co-investigators have received training from the principal investigator in how to obtain consent and answer questions that may arise during the consent process. The consent and letter have been written to comply with the requirement that they be written at a 5th grade reading level, evaluated by the Flesch–Kincaid readability test. In addition, subject will be asked to provide their understanding of what the study is about at the time of the consenting process. English is the primary language of all FDNY rescue workers, see Appendix.

Participant-related study information will be identified through the Patient Identification Number (PID) on all participant Case Report Forms (CRFs). Participant names or other personally-identifying information will not be used on any study documents. All study-related documents will be kept in double-locked, limited access areas at each study site. A log that links the names of participants to their PID numbers will also be kept under double locks separate from all other research records, accessible only to the study staff. Original source documents for individual participants will be maintained at the FDNY-BHS and will be accessible only to study staff.

### Case status

*WTC-AHR* will be defined as having a positive methacholine (PC_200_ < 16), or a positive bronchodilator response (by ATS/ERS guidelines with improvement of FEV_1_ by 12% and at least 200 mL) at least once post-9/11 [73, 74] and/or EMR diagnosis. *GERD* will be defined as: biopsy-proven erosive esophagitis LA grade C or D; stricture or Barrett’s esophagus on endoscopy; and/or esophageal acid exposure time > 6% on a pH or pH impedance study. GERD will also be defined on EMR diagnosis and/or PPIs, H_2_ blockers, antacid, or surface agent use [75]. *BE*, as a subset of GERD, will have any of the following additional inclusion criteria: biopsy-proven columnar epithelium lining ≥ 1 cm of the distal esophagus with intestinal metaplasia characterized with goblet cells on histology; diagnosis on EMR, Tables 2 and 3 [75]. The recruited participants will be consented prior to any research activity and measurement visit via REDCap software or in person.

**Table 2:**
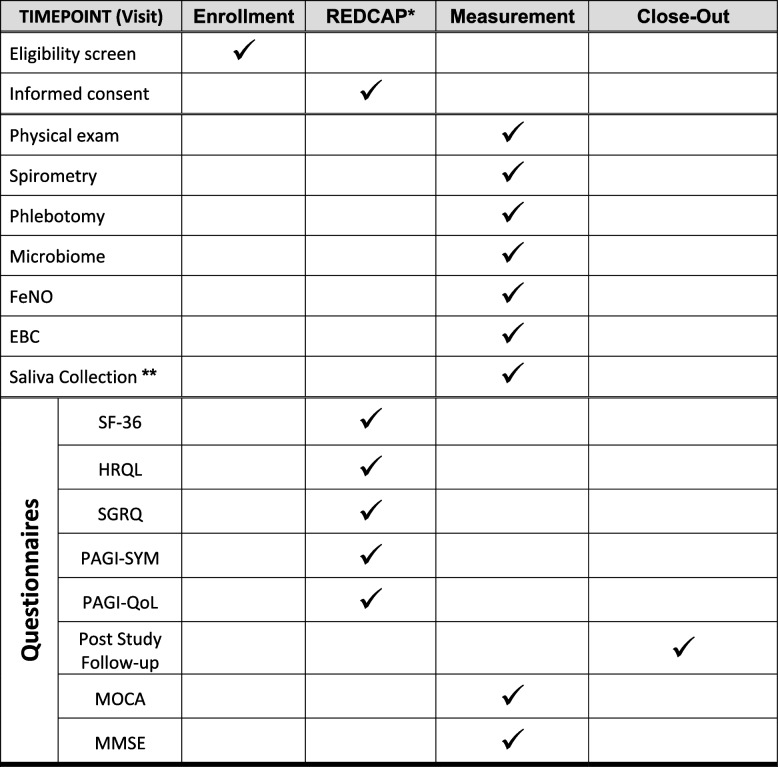
Schedule of study related activities

*FeNO* Fractional Exhaled Nitric Oxide, *EBC* Exhaled Breath Condensate, *SF-36* Short-Form 36, *HRQL* Health-Related Quality of Life, *SGRQ* St. George’s Respiratory Questionnaire, *PAGI-SYM* Patient Assessment of Upper Gastrointestinal Disorders- Symptoms Severity, *PAGI-QoL* Patient Assessment of Upper Gastrointestinal Disorders- Quality of Life, *MOCA* Montreal Cognitive Assessment, *MMSE* Mini Mental State Examination

^*^Consent and questionnaires may be obtained in person if subjects prefer

^**^Samples collected prior to in person visit

**Table 3:**
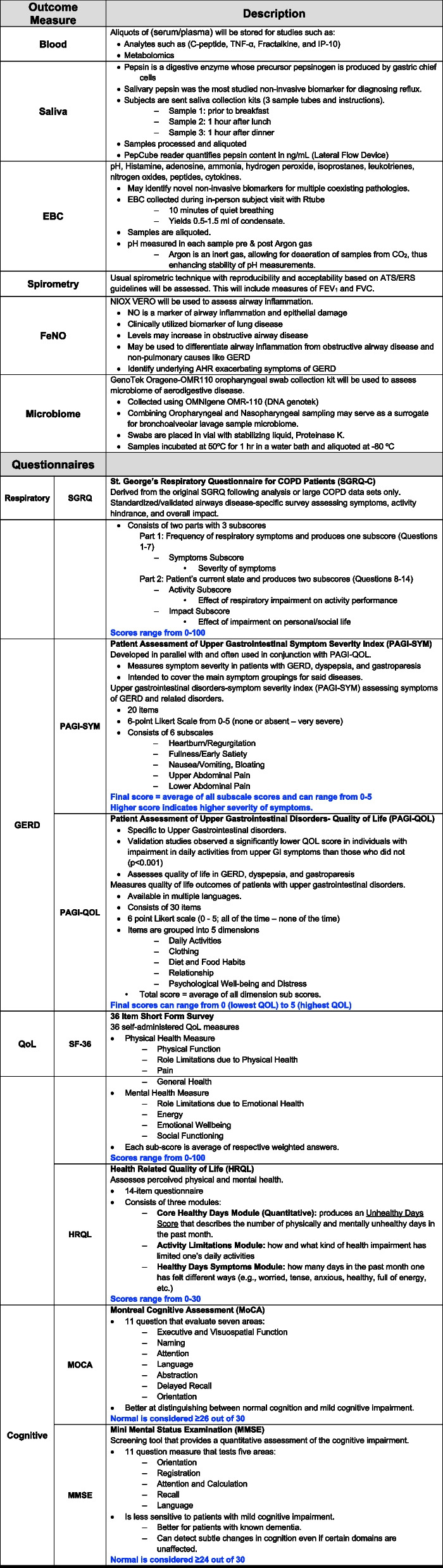
Endpoints of the BADBURN trial

*TNF-α* Tumor Necrosis Factor, *C peptide* Connecting peptide, *IP-10* Interferon-gamma-induced protein 10, *FEV1* Forced Expiratory Volume in 1 s, *EBC* Exhaled Breath Condensate, *FeNO* Fractional Exhaled Nitric Oxide, *SGRQ St.* George’s Respiratory Questionnaire, *GERD* Gastroesophageal Reflux Disease, *PAGI-SYM* Patient Assessment of Upper Gastrointestinal Disorders- Symptoms Severity, *PAGI-QOL* Patient Assessment of Upper Gastrointestinal Disorders- Quality of Life, *SF-36* Short-Form 36, *HRQL* Health-Related Quality of Life measures, *MOCA* Montreal Cognitive Assessment, *MMSE* Mini-Mental State Examination

### Measurement visit

Participant demographic information, medical history and medication history will be obtained. A physician will perform the physical examination, and verify that inclusion/exclusion criteria are met. Enrolled participants will undergo the following assessments.

### Blood sampling

After at least an 8 h fast, serum and plasma will be obtained, aliquoted and banked. Each stored specimen will be assigned a unique code to ensure proper identification and linkage to the respective participant. Aliquots from the fresh samples will be assayed for complete blood count (with differential) and chemistry panel. These data are already available for the banked samples. For all samples, lipid profile, metabolomics, and protein biomarker profiling will be performed [10, 28–30, 76, 77].

### Salivary pepsin assessment

30 mL sterile plastic tubes with 0.5 ml of 0.01 M citric acid, adjusted to a pH of 2.5 (RD Biomed Ltd., Hull, UK), will be used by the participants to collect saliva in the AM (prior to brushing teeth, drinking or eating), 1 h after finishing lunch, and 1 h after finishing dinner [78, 79]. Participants will be instructed to cough a few times prior to spitting into the tube to clear saliva from the back of the throat and then spit into the tube. The collected samples will be stored at 4 °C and analyzed within 2 days. Salivary Pepsin will be analyzed using Peptest (RD Biomed Ltd., Hull, UK) as previously described [79]. Briefly, plastic tubes will be centrifuged at 4,000 rpm for 5 min, and 80μL of supernatant will be added to 240μL of migration butter solution for 10 s. 80μL of the mixture will be added to the well of the Peptest, which contains two unique human monoclonal antibodies that detect and capture pepsin protein (specific to pepsin-3), with a lower limit of detection of 16 ng/mL and an upper limit of 500 ng/mL. A salivary pepsin level of ≥ 16 ng/mL will be considered positive. The sample will be processed in a Pepcube reader to quantify the pepsin concentration [78].

*Spirometry* will be assessed using a KoKo PFT spirometer (nSpire Health Inc)**,** and lung function assessment will be considered acceptable as per the ATS/ERS guidelines [80]. We will select the largest acceptable measures for electronic archiving. Each participant’s predicted percentage (%) will be calculated by NHANES III equations based on their age at examination, height, sex, and race [80, 81].

*FeNO* will be quantified using NIOX VERO® (Aerocrine) [82, 83]. Participants will be instructed to inhale to their total lung capacity via mouthpiece for 2–3 s. Then, they will exhale at a flow rate of 0.05L/second. The device will provide results in parts per billion (ppb).

*Exhaled breath condensate (EBC)* will be collected using RTubes (Respiratory Research, Inc., USA) [84]. Approximately 1-2 mL of EBC sample will be obtained after 10 min of quiet normal breathing [85]. *PH measurement.* EBC pH assay is extremely simple to perform, inexpensive, and robust, and can be easily processed on the day of collection [86]. EBC will be de-aerated of CO_2_ by bubbling free argon gas (350 ml/min) under a micro-pH reader (Orion PerpHecT micro-pH electrode) and stabilized pH will be recorded after approximately 3–5 min [87]. Aliquots are then stored at -80⁰C and thawed only once prior to histamine and biomarker assessment.

### Naso/oropharyngeal microbiome

#### Collection

Trained study team members will collect naso/oropharyngeal samples using commercially available kits (OMR-110 by DNA Genotek, Canada). Each naris will be swabbed in a circular fashion 10 times. The oropharyngeal sample will be collected by swabbing in the back of the throat in 10 circular motion to ensure sufficient swab collection. Each absorbent swab will be placed into a vial containing 1 mL of stabilizing liquid using aseptic technique. The sample will be treated with lyophilized Proteinase K, and incubated in the original vial at 50⁰C for 1 h in a water bath prior to aliquoting for long-term storage at -80⁰C.

### Quality of life, aerodigestive disease and end-organ effect questionnaires

*Gastrointestinal impact* will be assessed using with the Patient Assessment of Upper Gastrointestinal Disorders – Quality of Life (*PAGI-QoL*) and the Patient Assessment of Upper Gastrointestinal Disorders Symptom Severity Index (*PAGI-SYM*). Both questionnaires use a 6-point Likert scale (MAPI Research Trust) [88–91].

*Respiratory and QoL assessment* will utilize the Health-Related Quality of Life measures (*HRQL*) [92], St. George’s Respiratory Questionnaire (*SGRQ*), and the Short-form-36 (*SF-36*). HRQL assesses an individual's perceived physical and mental health. The SGRQ is a standardized, self-administered airways disease-specific questionnaire divided into three subscales- symptoms, activity, and impact [93]. SF-36 will capture supplemental information about their mental health, general health perception, emotional, and social role functioning [94].

*Cognition* will be assessed using the Montreal Cognitive Assessment (*MoCA*; version 8.1) and the Mini-Mental State Examination (*MMSE*). MMSE is a cognitive test used to evaluate early dementia [95, 96]. Combining MoCA and MMSE can improve diagnostic utility [97]. The MoCA will be administered by a trained/certified investigator. Members of our research team have completed MoCA training and certification through a validated MoCA cognition portal [98] (https://mocacognition.com/). Similar to the MoCA, the MMSE assesses orientation, memory, visuospatial and language domains. Additionally, the MMSE evaluates comprehension, reading and writing [99]. The PI will thoroughly review all scores.

### Outcomes

Levels of salivary pepsin, pH Levels from EBC, Histamine Concentration from EBC, Score on PAGI-QOL Questionnaire, Score on PAGI-SYM Questionnaire, Score on SGRQ-C Questionnaire, and SF-36.

### Power analysis

A sample size of 40 cases for each group of GERD, AHR, AHR/GERD overlap, BE, and non-GERD/non-AHR Controls (all will be subsets of AIM 1 *N =* 898 randomly selected cohort) achieves 80% power to detect difference as small as 0.78 SD with two-sample t-test at 0.01 significance level to account for multiple comparisons. This will allow us to achieve 80% power and significance of 0.05, based on prior studies with salivary pepsin test (personal communication with Dr. Peter Dettmar of Peptest), Fig. 2.

*Statistical Analysis* SPSS 28 (IBM) will facilitate database management and statistics. Continuous variables expressed as mean, standard deviation (SD) if normally distributed, and as median, inter-quartile range (IQR) if skewed. Two-sample t-test and ANOVA will compare continuous data. Count and proportions will summarize categorical data and Pearson- χ^2^ will compare categorical data. Multivariate binary logistic regression will estimate biomarker-disease relationship for case status as a binary outcome while adjusting for confounding. Cox proportional hazards model will evaluate the effects of biomarkers, smoking, and exposure on the hazard of developing WTC-GERD or BE over time. The maximum potential effectiveness of a biomarker will be calculated by Youden Index [100]. Goodness of fit, using the Hosmer–Lemeshow test. Survival curves compared by Log-rank test. Pearson χ^2^-test will compare SABA and LABA usage between GERD, AHR, AHR/GERD overlap, BE, and non-GERD or AHR controls. Significance will be assessed by *p <* 0.05 for all statistical tests. Graphs will be created using Prism (v.10, GraphPad Software).

#### Missing data

Variables with missing values in a small proportion of participants will be imputed using multiple imputation methods. To assess the missing at random assumption, we will evaluate the comparability between samples with missing data and those without. Sensitivity analysis will be performed by comparing the results obtained from the complete data analysis to the results obtained from multiple imputation.

### Model building

We have previously identified key biomarkers using a machine learning approach [10, 28–30]. We have further refined this analysis pipeline and will utilize this methodology to identify AHR, GERD, AHR/GERD overlap, and BE biomarkers**.** Specifically, we will utilize random forests (RF) of the filtered, normalized biomarkers. Models assessed via a modified hamming distance between variable importance rankings of models with identical hyper-parameters. A refined profile of the top 5% of important biomarkers by MDA will be included in a gradient-boosted tree model (xgboost package, R-Project) to build a classifier of AHR, GERD, AHR/GERD overlap, and BE. A random hyperparameter space search determined a final model that maximized AUC_ROC_.

We will also use linear mixed-effects models will be used to assess the temporal trend of biomarkers with time adjusting for confounders. The longitudinal biomarkers processes will be associated to risk of developing WTC-GERD/BE using the joint modeling technique [101]. The joint-modeling approach has become the primary method for analysis of longitudinal biomarker process and time-to-event outcome, and multiple R packages are available to implement the models. We will also consider a single index longitudinal model which enables us to reduce the dimensionality of multiple biomarkers and to evaluate joint effects of multiple biomarkers together to identify key risk factors. The single-index model incorporates longitudinal data to calculate hazard of each parameter as well as personalized dynamic risk for prognostication. Specifically, this will allow us to use a patient’s data from a single clinical exam to identify risk of GERD, AHR, overlap, or BE. Furthermore, this will allow the identification of false negatives and undertreated cases in the entire FDNY cohort.

## Discussion

PM exposure, a significant component of ambient and occupational exposures is a risk factor for aerodigestive disease (such as GERD and AHR) and is associated with approximately 7**-**million deaths annually [1–3, 11, 102–104]. GERD is the most prevalent gastrointestinal disorder in the US, with an estimate as high as 30% [66]. Globally, the prevalence of GERD ranges from 10–25%, with an increased risk in firefighters [52, 66]. GERD is an independent risk factor in the development of BE which can lead to malignancy [66]. 

Despite the similar risks, the understanding of the underlying pathophysiological interrelatedness between the aerodigestive diseases (AHR, GERD and BE) remains limited. Furthermore, GERD diagnosis and treatment has been invasive and costly. Therefore, our work is focused on identifying non-invasive biomarkers which may help identify at risk populations who may benefit from earlier intervention, targeted therapies and a further understanding of how their AHR is impacted by co-morbid GERD. The identification of non-invasive biomarkers of GERD/BE and the overlapping aerodigestive disease is crucial.

Our work will address the existing *knowledge gap* in aerodigestive overlap and validate biomarkers of WTC-aerodigestive disease. Biomarkers of BE may also identify individuals at risk for neoplastic disease. These findings may have broader implications for populations with GERD and PM exposure. In contrast to currently used invasive testing, noninvasive testing offers diagnostic utility with reduced risk and can direct future research into mechanisms/downstream effects. We also systematically studied biomarkers of GERD/BE and defined some of the lacunae in the non-invasive aerodigestive biomarker literature [105]. Therefore, our Case–Control Observational Study is designed to sample a broad biomarker profile, Table 3.

### Microbiome of the gut/lung axis

Asthma susceptibility is influenced by the gut microbiome [106–111]. Noninvasive collection sites that can approximate the pulmonary environment are of key interest. Studies have failed to show that the microbiomes of induced sputum were similar to the lung [112, 113]. Noninvasively collected oropharyngeal and nasopharyngeal swabs in conjunction could approximate the lung microbiome [114]. Research has revealed that the esophageal microbiome undergoes alteration in individuals with GERD, BE, and other motility disorders [115, 116]. Although these findings highlight the potential role of the microbiome studies in the diagnosis and therapeutic approaches for aerodigestive disease, further studies are needed and will be one of the key readouts planned in our study.

*EBC* analysis holds great promise in addressing unmet medical needs by expanding the portfolio of noninvasive assays for the multiple coexisting pathological mechanisms underlying respiratory disorders and GERD. Compounds identified in EBC include histamine, adenosine, ammonia, hydrogen peroxide, isoprostanes, leukotrienes, nitrogen oxides (NOx), peptides, cytokines, protons and various ions [85]. Histamine plays a vital role in digestion but elevated levels can contribute to the development of GERD [117, 118].

*Salivary pepsin* has been studied in several GERD biomarker studies [105]. Due to the overlap of various reflux symptoms with other GI pathologies, the diagnosis of GERD can be challenging. However, salivary pepsin test offers a simple and convenient way for detecting reflux through salivary sample collection, providing quick and non-invasive results. Compared to other diagnostic modalities, this approach is time-efficient and requires much less effort [119]. Moreover, pepsin measurements can identify pathologic reflux even in the absence of symptoms, and remain unaffected by the concurrent use of PPI. Several studies have demonstrated that pepsin detection in the sputum and/or saliva can be regarded as a sensitive, non-invasive method for the diagnosis of the proximal reflux of gastric contents, with a sensitivity ranged from 41.5% to 73% and high specificity of 86.2 to 98.2% [78, 79]. Despite these findings little is known about pepsin in the context of aerodigestive co-morbid disease.

*FeNO*, a biomarker of lung disease activity, will be a valuable measure in our population. FeNO is associated with airway hyperreactivity, and several studies demonstrated that FeNO is increased during obstructive exacerbations [120]. In our population with the aerodigestive overlap, FeNO levels can serve as an indicator of potential underlying AHR exacerbating symptoms of GERD. Thus, our work will also contribute to understand the role of FeNO in GERD, which remains inconclusive as only a limited number of studies have examined AHR/GERD [121, 122]. The detrimental impact of even once-weekly episodes of GERD on quality of life [123] highlighted the importance of assessing aerodigestive disease quality of life and disease activity, therefore we will quantify the effects of GERD on these aspects through a validated set of questionnaires that will assess QoL, GERD specific symptoms and also cognitive involvement.

Non-invasive biomarkers of GERD, BE, AHR, treatment efficacy, and severity of symptoms will also be assessed in serum. This will allow us to measure Tumor Necrosis Factor (TNF-α), C-peptide, Fractalkine and Interferon-gamma-induced Protein 10 (IP-10) in our case cohort study to validate our prior pilot study [1–3]. Serum samples will also be used to perform metabolomic profiling that will allow us to investigate metabolic correlates of aerodigestive disease. In addition, by validating serum biomarkers (proteins and metabolome) of GERD/BE, we seek to provide a biologically plausible target that enables early detection and facilitates therapeutic intervention in the PM exposed populations. Moreover, non-invasive phenotyping of WTC aerodigestive disease holds promise in improving the sensitivity and specificity of GERD diagnosis, enabling earlier identification of BE and facilitate the development of personalized therapy, thus to improve both the quality of life and overall health outcomes.

### Limitations and potential study concerns

We envision there are several limitations of our study. It is possible that no significant association exists between noninvasive biomarkers and aerodigestive diseases in the second decade after WTC exposure. The generalizability of our study could be impacted because the FDNY source cohort had no aerodigestive disease prior to 9/11 and had their serum samples banked within six months of 9/11, therefore making it less comparable to other cohorts. There may also be a subset of patients without history of GERD, but could still receive a clinical diagnosis of GERD based on questionnaires and/or elevated pepsin/biomarkers. For these patients, further follow-up with a gastroenterologist will be recommended. Additionally, we may use FeNO levels to identify the potential underlying AHR exacerbating symptoms associated with GERD. We will also account for the potential risk of loss to follow-up regarding the completion of the questionnaires and attendance of the in-person visit.

Further investigation into the overlap of GERD/BE and AHR is envisioned to provide valuable insights in distinguishing disease phenotypes, demonstrating that biomarkers can predict GERD and/or BE. This work will have clinical implications for the diagnosis and treatment of WTC associated disease, as well as for the management of other patients in the WTC monitoring programs, and for the general population as intense PM exposures are occurring more frequently, for example through wild fire related PM. Our research will contribute to the development of a robust biomarker set with optimal explanatory power when applied to diverse cohorts.

### Supplementary Information

Supplementary Material 1.

## Data Availability

No datasets were generated or analysed during the current study.

## Abbreviations

AHR: Airway Hyper reactivity
BADBURN: Biomarkers of Airway Disease, Barrett’s and Underdiagnosed Reflux Noninvasively
BMI: Body Mass Index (kg/m^2^)
COPD: Chronic Obstructive Pulmonary Disease
DBP: Diastolic Blood Pressure
EAC: Esophageal adenocarcinomas
ECG: Electrocardiogram
FDNY: Fire Department of New York
FE_NO_: Fractional Exhaled Nitric Oxide
FEV_1_: Forced Expiratory Volume in 1 s
FVC: Forced Vital Capacity
HRQL: Health-Related Quality of Life
HP: Health Program
IRB: Internal Review Board
LI: Lung Injury
LLN: Lower Limit of Normal
LoCalMed: Low Calorie Mediterranean Diet
MetSyn: Metabolic Syndrome
MoCA: Montreal Cognitive Assessment
MMSE: Mini-Mental State Examination
MOP: Manual of Procedures
MND: MyNetDiary
N: Number
NIH: National Institutes of Health
NYU: New York University
OAD: Obstructive Airways Disease
PAGI-QOL: Patient Assessment of Upper Gastrointestinal Disorders- Quality of Life
PAGI-SYM: Patient Assessment of Upper Gastrointestinal Disorders- Symptoms Severity
PI: Principal Investigator
PM: Particulate Matter
PWV: Pulse Wave Velocity
QoL: Quality of Life
RCT: Randomized Clinical Trial
SBP: Systolic Blood Pressure
SF-36: Short-Form 36
SGRQ: St. George’s Respiratory Questionnaire
SOP: Standard Operating Procedure
US: United States
WTC: World Trade Center
9/11: September 11, 2001

## References

[1] Seo HS, Hong J, Jung J. Relationship of meteorological factors and air pollutants with medical care utilization for gastroesophageal reflux disease in urban area. World J Gastroenterol. 2020;26:6074–86. 33132656 10.3748/wjg.v26.i39.6074 PMC7584054

[2] Gaffney KF. Infant exposure to environmental tobacco smoke. J Nurs Scholarsh. 2001;33:343–7. 11775304 10.1111/j.1547-5069.2001.00343.x

[3] Haider SH, et al. Predictive biomarkers of gastroesophageal reflux disease and barrett’s esophagus in World Trade Center exposed firefighters: a 15 year longitudinal study. Sci Rep. 2018;8:3106. 29449669 10.1038/s41598-018-21334-9 PMC5814524

[4] Veerappan A, et al. World trade center-cardiorespiratory and vascular dysfunction: assessing the phenotype and metabolome of a murine particulate matter exposure model. Sci Rep. 2020;10:3130. 32081898 10.1038/s41598-020-58717-w PMC7035300

[5] Haider SH, et al. Multiomics of World Trade Center particulate matter-induced persistent airway hyperreactivity. Role of receptor for advanced glycation end products. Am J Respir Cell Mol Biol. 2020;63:219–33. 32315541 10.1165/rcmb.2019-0064OC PMC7397767

[6] Long NP, et al. High-throughput omics and statistical learning integration for the discovery and validation of novel diagnostic signatures in colorectal cancer. Int J Mol Sci. 2019;20:296. 30642095 10.3390/ijms20020296 PMC6358915

[7] Clementi EA, et al. Metabolic syndrome and air pollution: a narrative review of their cardiopulmonary effects. Toxics. 2019;7:6. 30704059 10.3390/toxics7010006 PMC6468691

[8] Kwon S, et al. Metabolic syndrome biomarkers of World Trade Center airway hyperreactivity: a 16-year prospective cohort study. Int J Environ Res Public Health. 2019;16:1486. 31035527 10.3390/ijerph16091486 PMC6539892

[9] Haider SH, et al. Receptor for advanced glycation end-products and environmental exposure related obstructive airways disease: a systematic review. Eur Respir Rev. 2019;28:180096. 30918021 10.1183/16000617.0096-2018 PMC7006869

[10] Crowley G, et al. Assessing the protective metabolome using machine learning in World Trade Center particulate exposed firefighters at risk for lung injury. Sci Rep. 2019;9:11939. 31481674 10.1038/s41598-019-48458-w PMC6722247

[11] Haider SH, et al. Predictive biomarkers of gastroesophageal reflux disease and Barrett’s esophagus in World Trade Center exposed firefighters: a 15 year longitudinal study. Sci Rep-Uk. 2018;8:3106. 10.1038/s41598-018-21334-9 PMC5814524 29449669

[12] de la Hoz RE, et al. Increased pulmonary artery diameter is associated with reduced FEV1 in former World Trade Center workers. Clin Respir J. 2019;13:614–23. 31347281 10.1111/crj.13067 PMC6783324

[13] Singh A, et al. Predictors of asthma/COPD overlap in FDNY firefighters with world trade center dust exposure: a longitudinal study. Chest. 2018;154:1301–10. 30028968 10.1016/j.chest.2018.07.002 PMC6289858

[14] Mikhail M, et al. Non-cardiac chest pain: a review of environmental exposure-associated comorbidities and biomarkers. EMJ Gastroenterol. 2018;7:103–12. 30774967 10.33590/emjgastroenterol/10313895 PMC6375490

[15] Beattie J, et al. Zika virus-associated Guillain-Barre syndrome in a returning US traveler. Infect Dis Clin Prac. 2018;26:E80–4. 10.1097/IPC.0000000000000654 PMC6433380 30923438

[16] Stream S, Nolan A, Kwon S, Constable C. Factors associated with combined do-not-resuscitate and do-not-intubate orders: a retrospective chart review at an urban tertiary care center. Resuscitation. 2018;130:1–5. 29935341 10.1016/j.resuscitation.2018.06.020 PMC6481924

[17] Hena KM, et al. Clinical course of sarcoidosis in World Trade Center-exposed firefighters. Chest. 2018;153:114–23. 29066387 10.1016/j.chest.2017.10.014 PMC6026251

[18] Zeig-Owens R, et al. Blood leukocyte concentrations, FEV1 decline, and airflow limitation a 15-year longitudinal study of World Trade Center-exposed firefighters. Ann Am Thorac Soc. 2018;15:173–83. 29099614 10.1513/AnnalsATS.201703-276OC PMC5802620

[19] Crowley G, et al. Metabolomics of world trade center-lung injury: a machine learning approach (vol 5, e000274, 2018). Bmj Open Respir Res. 2018;5:e000274. 30233801 10.1136/bmjresp-2017-000274 PMC6135464

[20] Lee YI, et al. Fluid resuscitation-associated increased mortality and inflammatory cytokine expression in murine polymicrobial sepsis. J Clin Transl Sci. 2017;1:265–6. 29657863 10.1017/cts.2017.15 PMC5890308

[21] Vossbrinck M, et al. Post-9/11/2001 lung function trajectories by sex and race in World Trade Center-exposed New York City emergency medical service workers. Occup Environ Med. 2017;74:200–3. 27810938 10.1136/oemed-2016-103619 PMC5573813

[22] Caraher EJ, et al. Receptor for advanced glycation end-products and World Trade Center particulate induced lung function loss: a case-cohort study and murine model of acute particulate exposure. PLoS ONE. 2017;12:e0184331. 28926576 10.1371/journal.pone.0184331 PMC5604982

[23] Aldrich TK, et al. Bronchial reactivity and lung function after World Trade Center exposure. Chest. 2016;150:1333–40. 27445092 10.1016/j.chest.2016.07.005 PMC6026231

[24] Kwon S, Crowley G, Haider SH, Zhang L, Nolan A. Nephroprotective strategies in septic shock: the VANISH trial. J Thorac Dis. 2016;8:E1508–10. 28066645 10.21037/jtd.2016.11.44 PMC5179465

[25] Zeig-Owens R, et al. Biomarkers of patient intrinsic risk for upper and lower airway injury after exposure to the World Trade Center atrocity. Am J Ind Med. 2016;59:788–94. 27582481 10.1002/ajim.22643 PMC5573814

[26] Zhang L, et al. Air pollution and lung function loss: the importance of metabolic syndrome. Austin J Pulm Respir Med. 2016;3:1043. 27868109 PMC5114002

[27] Weiden MD, et al. Biomarkers of World Trade Center particulate matter exposure: physiology of distal airway and blood biomarkers that predict FEV(1) decline. Semin Respir Crit Care Med. 2015;36:323–33. 26024341 10.1055/s-0035-1547349 PMC4755483

[28] Caraher EJ, et al. Receptor for advanced glycation end-products and World Trade Center particulate induced lung function loss: a case-cohort study and murine model of acute particulate exposure. Plos One. 2017;12:e0184331. 28926576 10.1371/journal.pone.0184331 PMC5604982

[29] Crowley G, et al. Metabolite and biomarker predictors of World Trade Center-lung injury: an integrated multiplatform machine learning approach. Am J Respir Crit Care Med. 2018;197:A2588.

[30] Crowley G, et al. Metabolomics of World Trade Center-Lung Injury: a machine learning approach. Bmj Open Respir Res. 2018;5:e000274. 30233801 10.1136/bmjresp-2017-000274 PMC6135464

[31] Kwon S, et al. Metabolic syndrome biomarkers of World Trade Center airway hyperreactivity: a 16-year prospective cohort study. Int J Environ Res Public Health. 2019;16:1486. 31035527 10.3390/ijerph16091486 PMC6539892

[32] Lam R, et al. Synergistic effect of WTC-particulate matter and lysophosphatidic acid exposure and the role of RAGE: in-vitro and translational assessment. Int J Environ Res Public Health. 2020;17:4318. 32560330 10.3390/ijerph17124318 PMC7344461

[33] Lee YI, et al. Predictors of acute hemodynamic decompensation in early sepsis: an observational study. J Clin Med Res. 2016;8:575–81. 27429677 10.14740/jocmr2597w PMC4931802

[34] Aldrich TK, et al. Lung function trajectories in World Trade Center-exposed New York City firefighters over 13 years: the roles of smoking and smoking cessation. Chest. 2016;149:1419–27. 26836912 10.1016/j.chest.2015.10.067 PMC6026237

[35] Weiden MD, Zeig-Owens R, Singh A, Schwartz T, Liu Y, Vaeth B, Nolan A, Cleven KL, Hurwitz K, Beecher S, Prezant DJ. Pre-COVID-19 Lung Function and Other Risk Factors for Severe COVID-19 in First Responders. ERJ Open Res. 2020;00610–2020. 10.1183/23120541.00610-2020. 10.1183/23120541.00610-2020 PMC7607970 33527077

[36] Aldrich TK, et al. Lung function in rescue workers at the World Trade Center after 7 years. N Engl J Med. 2010;362:1263–72. 20375403 10.1056/NEJMoa0910087 PMC4940972

[37] Aldrich TK, et al. Longitudinal pulmonary function in newly hired, non-World Trade Center-exposed fire department City of New York firefighters: the first 5 years. Chest. 2013;143:791–7. 23188136 10.1378/chest.12-0675 PMC3590887

[38] Banauch GI, et al. Bronchial hyperreactivity and other inhalation lung injuries in rescue/recovery workers after the World Trade Center collapse. Crit Care Med. 2005;33:S102-106. 15640671 10.1097/01.CCM.0000151138.10586.3A

[39] Banauch GI, Dhala A, Prezant DJ. Pulmonary disease in rescue workers at the World Trade Center site. Curr Opin Pulm Med. 2005;11:160–8. 15699790 10.1097/01.mcp.0000151716.96241.0a

[40] de la Hoz RE, et al. Reflux symptoms and disorders and pulmonary disease in former World Trade Center rescue and recovery workers and volunteers. J Occup Environ Med. 2008;50:1351–4. 19092489 10.1097/JOM.0b013e3181845f9b

[41] Prezant DJ, et al. Cough and bronchial responsiveness in firefighters at the World Trade Center site. N Engl J Med. 2002;347:806–15. 12226151 10.1056/NEJMoa021300

[42] Li J, et al. Gastroesophageal reflux symptoms and comorbid asthma and posttraumatic stress disorder following the 9/11 terrorist attacks on World Trade Center in New York City. Am J Gastroenterol. 2011;106:1933–41. 21894225 10.1038/ajg.2011.300

[43] Webber MP, et al. Trends in respiratory symptoms of firefighters exposed to the world trade center disaster: 2001–2005. Environ Health Perspect. 2009;117:975–80. 19590693 10.1289/ehp.0800291 PMC2702416

[44] Dent J, El-Serag HB, Wallander MA, Johansson S. Epidemiology of gastro-oesophageal reflux disease: a systematic review. Gut. 2005;54:710–7. 15831922 10.1136/gut.2004.051821 PMC1774487

[45] Savarino E, et al. Advances in the physiological assessment and diagnosis of GERD. Nat Rev Gastroenterol Hepatol. 2017;14:665. 28951582 10.1038/nrgastro.2017.130

[46] Shaheen NJ, et al. The burden of gastrointestinal and liver diseases, 2006. Am J Gastroenterol. 2006;101:2128–38. 16848807 10.1111/j.1572-0241.2006.00723.x

[47] Yip J, et al. FDNY and 9/11: clinical services and health outcomes in World Trade Center-exposed firefighters and EMS workers from 2001 to 2016. Am J Ind Med. 2016;59:695–708. 27427498 10.1002/ajim.22631

[48] Lagergren J, Bergström R, Lindgren A, Nyrén O. Symptomatic gastroesophageal reflux as a risk factor for esophageal adenocarcinoma. N Engl J Med. 1999;340:825–31. 10080844 10.1056/NEJM199903183401101

[49] Hvid-Jensen F, Pedersen L, Drewes AM, Sørensen HT, Funch-Jensen P. Incidence of adenocarcinoma among patients with Barrett’s esophagus. N Engl J Med. 2011;365:1375–83. 21995385 10.1056/NEJMoa1103042

[50] Lagergren J, Bergstrom R, Lindgren A, Nyren O. Symptomatic gastroesophageal reflux as a risk factor for esophageal adenocarcinoma. N Engl J Med. 1999;340:825–31. 10080844 10.1056/NEJM199903183401101

[51] Mody R, et al. Comparison of health care resource utilization and costs among patients with GERD on once-daily or twice-daily proton pump inhibitor therapy. Clinicoecon Outcomes Res. 2013;5:161–9. 23637544 10.2147/CEOR.S41189 PMC3639021

[52] Jang SH, Ryu HS, Choi SC, Lee SY. Psychological factors influence the gastroesophageal reflux disease (GERD) and their effect on quality of life among firefighters in South Korea. Int J Occup Environ Health. 2016;22:315–20. 27691373 10.1080/10773525.2016.1235675 PMC5137555

[53] Francis DO, et al. High economic burden of caring for patients with suspected extraesophageal reflux. Am J Gastroenterol. 2013;108:905–11. 23545710 10.1038/ajg.2013.69

[54] Ghisa M, et al. Updates in the field of non-esophageal gastroesophageal reflux disorder. Expert Rev Gastroenterol Hepatol. 2019;13:827–38. 31322443 10.1080/17474124.2019.1645593

[55] Havemann BD, Henderson CA, El-Serag HB. The association between gastro-oesophageal reflux disease and asthma: a systematic review. Gut. 2007;56:1654–64. 17682001 10.1136/gut.2007.122465 PMC2095717

[56] Clouston SAP, et al. Proton pump inhibitors and the risk of severe cognitive impairment: the role of posttraumatic stress disorder. Alzheimers Dement (N Y). 2017;3:579–83. 29124117 10.1016/j.trci.2017.08.007 PMC5671627

[57] Choi HG, et al. Associations between proton pump inhibitors and Alzheimer’s disease: a nested case–control study using a Korean nationwide health screening cohort. Alzheimers Res Ther. 2022;14:91. 10.1186/s13195-022-01032-5 PMC9248149 35773740

[58] Kawar N, et al. Salivary microbiome with gastroesophageal reflux disease and treatment. Sci Rep. 2021;11:188. 33420219 10.1038/s41598-020-80170-y PMC7794605

[59] Patel V, Ma S, Yadlapati R. Salivary biomarkers and esophageal disorders. Dis Esophagus. 2022;35:doac018. 35397479 10.1093/dote/doac018 PMC13375636

[60] Du X, et al. The diagnostic value of pepsin detection in saliva for gastro-esophageal reflux disease: a preliminary study from China. BMC Gastroenterol. 2017;17:107. 29041918 10.1186/s12876-017-0667-9 PMC5645897

[61] Schenck JF, Zimmerman EA. High-field magnetic resonance imaging of brain iron: birth of a biomarker? NMR Biomed. 2004;17:433–45. 15523705 10.1002/nbm.922

[62] Naveed B, et al. Metabolic syndrome biomarkers predict lung function impairment: a nested case-control study. Am J Respir Crit Care Med. 2012;185:392–9. 22095549 10.1164/rccm.201109-1672OC PMC3297095

[63] Nolan A, et al. Inflammatory biomarkers predict airflow obstruction after exposure to World Trade Center dust. Chest. 2012;142:412–8. 21998260 10.1378/chest.11-1202 PMC3425337

[64] Tsukiji J, et al. Lysophosphatidic acid and apolipoprotein A1 predict increased risk of developing World Trade Center-lung injury: a nested case-control study. Biomarkers. 2014;19:159–65. 24548082 10.3109/1354750X.2014.891047 PMC4306444

[65] Weiden MD, et al. Cardiovascular biomarkers predict susceptibility to lung injury in World Trade Center dust-exposed firefighters. Eur Respir J. 2013;41:1023–30. 22903969 10.1183/09031936.00077012 PMC3642231

[66] Richter JE, Rubenstein JH. Presentation and epidemiology of gastroesophageal reflux disease. Gastroenterology. 2018;154:267–76. 28780072 10.1053/j.gastro.2017.07.045 PMC5797499

[67] Lim SW, et al. Management of asymptomatic erosive esophagitis: an e-mail survey of physician’s opinions. Gut Liver. 2013;7:290–4. 23710309 10.5009/gnl.2013.7.3.290 PMC3661960

[68] Lu CL. Silent gastroesophageal reflux disease. J Neurogastroenterol Motil. 2012;18:236–8. 22837870 10.5056/jnm.2012.18.3.236 PMC3400810

[69] Ronkainen J, et al. Prevalence of Barrett’s esophagus in the general population: an endoscopic study. Gastroenterology. 2005;129:1825–31. 16344051 10.1053/j.gastro.2005.08.053

[70] Ashrafian H, Neubauer S. Metabolomic profiling of cardiac substrate utilization: fanning the flames of systems biology? Circulation. 2009;119:1700–2. 19349333 10.1161/CIRCULATIONAHA.109.849919

[71] Weiden MD, et al. Obstructive airways disease with air trapping among firefighters exposed to World Trade Center dust. Chest. 2010;137:566–74. 19820077 10.1378/chest.09-1580 PMC2832867

[72] Kwon S, et al. Dynamic metabolic risk profiling of world trade center lung disease: a longitudinal cohort study. Am J Respir Crit Care Med. 2021;204:1035–47. 34473012 10.1164/rccm.202006-2617OC PMC8663002

[73] O’Byrne PM, Inman MD. Airway hyperresponsiveness. Chest. 2003;123:411S-416S. 12629006 10.1378/chest.123.3_suppl.411S

[74] Pellegrino R, et al. Interpretative strategies for lung function tests. Eur Respir J. 2005;26:948–68. 16264058 10.1183/09031936.05.00035205

[75] Weakley J, et al. Trends in respiratory diagnoses and symptoms of firefighters exposed to the World Trade Center disaster: 2005–2010. Prev Med. 2011;53:364–9. 21930151 10.1016/j.ypmed.2011.09.001

[76] Nolan A, et al. Inflammatory biomarkers predict airflow obstruction after exposure to World Trade Center dust. Chest. 2012;142:412–8. 21998260 10.1378/chest.11-1202 PMC3425337

[77] Breier M, et al. Targeted metabolomics identifies reliable and stable metabolites in human serum and plasma samples. PLoS ONE. 2014;9:e89728. 24586991 10.1371/journal.pone.0089728 PMC3933650

[78] Du X, et al. The diagnostic value of pepsin detection in saliva for gastro-esophageal reflux disease: a preliminary study from China. BMC Gastroenterol. 2017;17:107. 29041918 10.1186/s12876-017-0667-9 PMC5645897

[79] Hayat JO, et al. Pepsin in saliva for the diagnosis of gastro-oesophageal reflux disease. Gut. 2015;64:373–80. 24812000 10.1136/gutjnl-2014-307049

[80] Wanger J, et al. Standardisation of the measurement of lung volumes. Eur Respir J. 2005;26:511–22. 16135736 10.1183/09031936.05.00035005

[81] Herbert R, et al. The World Trade Center disaster and the health of workers: five-year assessment of a unique medical screening program. Environ Health Perspect. 2006;114:1853–8. 17185275 10.1289/ehp.9592 PMC1764159

[82] American Thoracic Society, European Respiratory Society. ATS/ERS recommendations for standardized procedures for the online and offline measurement of exhaled lower respiratory nitric oxide and nasal nitric oxide, 2005. Am J Respir Crit Care Med. 2005;171:912–30. 15817806 10.1164/rccm.200406-710ST

[83] Dweik RA, et al. An official ATS clinical practice guideline: interpretation of exhaled nitric oxide levels (FENO) for clinical applications. Am J Respir Crit Care Med. 2011;184:602–15. 21885636 10.1164/rccm.9120-11ST PMC4408724

[84] Hunt J. Exhaled breath condensate: an overview. Immunol Allergy Clin. 2007;27:587–96. 10.1016/j.iac.2007.09.001 PMC2170898 17996577

[85] Horvath I, et al. Exhaled breath condensate: methodological recommendations and unresolved questions. Eur Respir J. 2005;26:523–48. 16135737 10.1183/09031936.05.00029705

[86] Vaughan J, et al. Exhaled breath condensate pH is a robust and reproducible assay of airway acidity. Eur Respir J. 2003;22:889–94. 14680074 10.1183/09031936.03.00038803

[87] Prieto L, Orosa B, Barato D, Marin J. The effect of different periods of argon deaeration on exhaled breath condensate pH. J Asthma. 2011;48:319–23. 21385108 10.3109/02770903.2011.560321

[88] De La Loge C, et al. Responsiveness and interpretation of a quality of life questionnaire specific to upper gastrointestinal disorders. Clin Gastroenterol Hepatol. 2004;2:778–86. 15354278 10.1016/S1542-3565(04)00349-0

[89] Rentz AM, et al. Development and psychometric evaluation of the patient assessment of upper gastrointestinal symptom severity index (PAGI-SYM) in patients with upper gastrointestinal disorders. Qual Life Res. 2004;13:1737–49. 15651544 10.1007/s11136-004-9567-x

[90] de la Loge C, et al. Cross-cultural development and validation of a patient self-administered questionnaire to assess quality of life in upper gastrointestinal disorders: the PAGI-QOL. Qual Life Res. 2004;13:1751–62. 15651545 10.1007/s11136-004-8751-3

[91] Wyrwich KW, et al. Validation of the PAGI-SYM and PAGI-QOL among healing and maintenance of erosive esophagitis clinical trial participants. Qual Life Res. 2010;19:551–64. 20195905 10.1007/s11136-010-9620-x

[92] Yin S, Njai R, Barker L, Siegel PZ, Liao Y. Summarizing health-related quality of life (HRQOL): development and testing of a one-factor model. Popul Health Metr. 2016;14:22. 27408606 10.1186/s12963-016-0091-3 PMC4940947

[93] Jones PW, Quirk FH, Baveystock CM. The St George’s respiratory questionnaire. Respir Med. 1991;85 Suppl B:25–31. discussion 33-27. 1759018 10.1016/S0954-6111(06)80166-6

[94] Brazier JE, et al. Validating the SF-36 health survey questionnaire: new outcome measure for primary care. BMJ. 1992;305:160–4. 1285753 10.1136/bmj.305.6846.160 PMC1883187

[95] Arevalo-Rodriguez I, et al. Mini-Mental State Examination (MMSE) for the early detection of dementia in people with mild cognitive impairment (MCI). Cochrane Database Syst Rev. 2021;7:Cd010783. 34313331 10.1002/14651858.CD010783.pub3 PMC8406467

[96] Nasreddine ZS, et al. The Montreal Cognitive Assessment, MoCA: a brief screening tool for mild cognitive impairment. J Am Geriatr Soc. 2005;53:695–9. 15817019 10.1111/j.1532-5415.2005.53221.x

[97] Flicker L, Logiudice D, Carlin JB, Ames D. The predictive value of dementia screening instruments in clinical populations. Int J Geriatr Psychiatry. 1997;12:203–9. 9097213 10.1002/(SICI)1099-1166(199702)12:2<203::AID-GPS603>3.0.CO;2-W

[98] Nasreddine ZS. MoCA test mandatory training and certification: what is the purpose? J Am Geriatr Soc. 2020;68:444–5. 31792923 10.1111/jgs.16267

[99] Shigemori K, Ohgi S, Okuyama E, Shimura T, Schneider E. The factorial structure of the Mini-Mental State Examination (MMSE) in Japanese dementia patients. BMC Geriatr. 2010;10:36. 20534132 10.1186/1471-2318-10-36 PMC2903593

[100] Ruopp MD, Perkins NJ, Whitcomb BW, Schisterman EF. Youden index and optimal cut-point estimated from observations affected by a lower limit of detection. Biom J Biometrische Zeitschrift. 2008;50:419–30. 18435502 10.1002/bimj.200710415 PMC2515362

[101] Rizopoulos D. JM: an R package for the joint modelling of longitudinal and time-to-event data. J Stat Softw. 2010;35:1–33.21603108 10.18637/jss.v035.i09

[102] Coppeta L, Pietroiusti A, Magrini A, Somma G, Bergamaschi A. Prevalence and characteristics of functional dyspepsia among workers exposed to cement dust. Scand J Work Env Hea. 2008;34:396–402. 10.5271/sjweh.1275 18853070

[103] Joo YH, Lee SS, Han KD, Park KH. Association between chronic laryngitis and particulate matter based on the Korea National Health and Nutrition Examination Survey 2008–2012. Plos One. 2015;10:e0133180. 26177353 10.1371/journal.pone.0133180 PMC4503512

[104] Organization WH. 9 out of 10 people worldwide breathe polluted air, but more countries are taking action. 2018.

[105] Farooqi MS, et al. Noninvasive, MultiOmic, and multicompartmental biomarkers of reflux disease: a systematic review. Gastro Hep Adv. 2023;2:608–20. 38009162 10.1016/j.gastha.2023.01.014 PMC10673619

[106] Abrahamsson TR, et al. Low diversity of the gut microbiota in infants with atopic eczema. J Allergy Clin Immun. 2012;129:434-U244. 22153774 10.1016/j.jaci.2011.10.025

[107] Bruzzese E, et al. Disrupted intestinal microbiota and intestinal inflammation in children with cystic fibrosis and its restoration with Lactobacillus GG: a randomised clinical trial. Plos One. 2014;9:e87796. 24586292 10.1371/journal.pone.0087796 PMC3929570

[108] Fagundes CT, et al. Transient TLR activation restores inflammatory response and ability to control pulmonary bacterial infection in germfree mice. J Immunol. 2012;188:1411–20. 22210917 10.4049/jimmunol.1101682

[109] Inagaki H, Suzuki K, Nomoto K, Yoshikai Y. Increased susceptibility to primary infection with Listeria monocytogenes in germfree mice may be due to lack of accumulation of L-selectin(+) CD44(+) T cells in sites of inflammation. Infect Immun. 1996;64:3280–7. 8757865 10.1128/iai.64.8.3280-3287.1996 PMC174219

[110] Clarke TB. Early innate immunity to bacterial infection in the lung is regulated systemically by the commensal microbiota via nod-like receptor ligands. Infect Immun. 2014;82:4596–606. 25135683 10.1128/IAI.02212-14 PMC4249320

[111] Segal LN, Rom WN, Weiden MD. Lung microbiome for clinicians New discoveries about bugs in healthy and diseased lungs. Ann Am Thorac Soc. 2014;11:108–16. 24460444 10.1513/AnnalsATS.201310-339FR PMC3972985

[112] An SQ, Warris A, Turner S. Microbiome characteristics of induced sputum compared to bronchial fluid and upper airway samples. Pediatr Pulmonol. 2018;53:921–8. 29727521 10.1002/ppul.24037

[113] Millares L, et al. The respiratory microbiome in bronchial mucosa and secretions from severe IgE-mediated asthma patients. BMC Microbiol. 2017;17:20. 28103814 10.1186/s12866-017-0933-6 PMC5248442

[114] Marsh RL, et al. The microbiota in bronchoalveolar lavage from young children with chronic lung disease includes taxa present in both the oropharynx and nasopharynx. Microbiome. 2016;4:37. 27388563 10.1186/s40168-016-0182-1 PMC4936249

[115] Lv J, et al. Alteration of the esophageal microbiota in Barrett’s esophagus and esophageal adenocarcinoma. World J Gastroenterol. 2019;25:2149–61. 10.3748/wjg.v25.i18.2149 PMC6526156 31143067

[116] Corning B, Copland AP, Frye JW. The esophageal microbiome in health and disease. Curr Gastroenterol Rep. 2018;20:39. 30069679 10.1007/s11894-018-0642-9

[117] Prinz C, Zanner R, Gratzl M. Physiology of gastric enterochromaffin-like cells. Annu Rev Physiol. 2003;65:371–82. 12221195 10.1146/annurev.physiol.65.092101.142205

[118] Håkanson R, et al. The biology and physiology of the ECL cell. Yale J Biol Med. 1994;67:123–34. 7502521 PMC2588926

[119] Boulton KHA, Fisher J, Woodcock AD, Dettmar PW. Pepsin as a biomarker for self-diagnosing reflux associated symptoms in UK and USA individuals. Ann Esophagus. 2021;4:23. 10.21037/aoe-20-79

[120] Maziak W, et al. Exhaled nitric oxide in chronic obstructive pulmonary disease. Am J Respir Crit Care Med. 1998;157:998–1002. 9517624 10.1164/ajrccm.157.3.97-05009

[121] Silvestri M, et al. Correlations between exhaled nitric oxide levels, blood eosinophilia, and airway obstruction reversibility in childhood asthma are detectable only in atopic individuals. Pediatr Pulmonol. 2003;35:358–63. 12687592 10.1002/ppul.10264

[122] Kowal K, Bodzenta-Lukaszyk A, Zukowski S. Exhaled nitric oxide in evaluation of young adults with chronic cough. J Asthma. 2009;46:692–8. 19728207 10.1080/02770900903056187

[123] Ronkainen J, et al. Gastro-oesophageal reflux symptoms and health-related quality of life in the adult general population - the Kalixanda study. Aliment Pharm Ther. 2006;23:1725–33. 10.1111/j.1365-2036.2006.02952.x 16817916

