# Supplementary material for: Biomarkers of Airway Disease, Barrett’s and Underdiagnosed Reflux Noninvasively (BAD-BURN) in World Trade Center exposed firefighters: a case–control observational study protocol
Source: BMC Gastroenterol. 2024 Aug 9;24:255. doi: 10.1186/s12876-024-03294-9 (PMC11312152; doi:10.1186/s12876-024-03294-9)
Supplement: Supplementary file 1 — Supplementary Material 1. [file 12876_2024_3294_MOESM1_ESM.pdf]

NYU Langone Health

Research Subject Informed Consent Form

**Title of Study:**	AERODIGESTIVE DISEASE IN THE WORLD TRADE CENTER EXPOSED FDNY COHORT: a Single Center Observational Study of Biomarkers of Airway Disease, Barrett's and Underdiagnosed Reflux Noninvasively **(BADBURN)**<br><br>**s21-00679**
**Principal Investigator:**	**Anna Nolan, MD, MS**<br>Professor, Department of Medicine and Environmental Medicine<br>NYU School of Medicine<br>462 1st Avenue<br>OBV A612 and Lab 16-North Room 20<br>646-501-6783(Lab)<br>anna.nolan@med.nyu.edu
**Emergency Contact:**	**Anna Nolan, MD**<br>646-501-6783

1. About volunteering for this research study

You are being invited to take part in a research study. Your participation is voluntary which means you can choose whether or not you want to take part in this study.

People who agree to take part in research studies are called "subjects" or "research subjects". These words are used throughout this consent form. Before you can make your decision, you will need to know what the study is about, the possible risks and benefits of being in this study, and what you will have to do in this study. You may also decide to discuss this study and this form with your family, friends, or doctor. If you have any questions about the study or about this form, please ask us. If you decide to take part in this study, you must sign this form.

We will give you a copy of this form signed by you for you to keep.

2. What is the purpose of this study?

We invite you to take part in a research study because:

1. You have World Trade Center-aerodigestive conditions. WTC-aerodigestive disease includes participants that have either WTC associated Airway Hyperreactivity (AHR), Gastroesophageal reflux disease (GERD), Barrett's esophagus or overlap of these conditions).
2. You were exposed to the WTC site but didn't develop WTC-aerodigestive disease.

Studies show that biomarkers which relate to the function of your body's cells may be important to the development of WTC- associated diseases. We would like to measure these biomarkers in samples that we will obtain from you. We hope to find which biomarkers are clinically useful in managing the health of those in WTC monitoring programs.

3. How long will I be in the study? How many other people will be in the study?

Study participants will be enrolled in the study for a total of 2 years. We expect that 200 people will join our study. All research subjects will be current or former members of the Fire Department of New York (FDNY) and have been WTC-exposed.

4. What will I be asked to do in the study?

If you agree to the study, you will need to participate in the following activities:

Screening/enrollment (this will last no more than 15 min)

- We will review your medical history and medications to make sure you qualify for the study.
- We will schedule your study visits if you're eligible to take part.
- We will give you instructions needed to prepare for your first study visit
- Read and sign this document if you agree to take part in the study.

Measurement Visit (this visit will last no more than 1 hour)

- This visit will occur within 1 year of the Screening Visit.
- Verify inclusion/exclusion criteria
- Obtain demographic information, medical history, and medication history
- Undergo a physical exam
- Collect specimens and the following measures.
    - **Blood:** We will draw approximately 30 mLs (2 tablespoons) of blood to perform several tests including Complete Blood Count with differential, Complete Metabolic, and lipid panel.
    - **Spirometry:** used to measure airflow obstruction often seen in WTC-LI. You will be given a sterile mouthpiece which is connected via a tube to the spirometer machine. You will be asked to inhale as deeply as you can for roughly 2-3 seconds, and then to exhale forcefully and quickly for about 5-6 seconds. The test is often repeated 3-4 times for consistency. The entirety of this testing takes only about 5 minutes, and rarely causes harm or discomfort.
    - **FeNO measurement:** used to measure inflammation in the airways often seen in WTC-LI. You will be given a sterile mouthpiece attached to the FeNO machine, asked to inhale as deeply as you can for roughly 2-3 seconds, and then to exhale slowly for about 10 seconds. This test takes only about 1 minute and causes no harm or discomfort.
    - **Saliva.** You will be asked to collect your saliva in a sterile tube that will be provided in the AM (prior to brushing, drinking or eating), 1h after finishing lunch, and dinner. You will be instructed to cough a few times prior to spitting into the tube to clear saliva from the back of the throat and then spit into the tube. You will be provided these tubes and will do this in your home. Samples will be stored in your refrigerator no more than 2 days prior to your visit. You will then bring the samples to your visit for us to process (these will be mailed to use and you may return them to us at the time of your measurement visit).
    - **Exhaled breath condensate (EBC)** will be collected in 10 min of quiet breathing into a chilled collecting tube.
    - **Microbiome sampling:** Naso/oropharyngeal sample will be obtained using a swab that will be placed into your nose and back of your mouth similar to a COVID-19 test

- You will also be given **7 questionnaires** that will assess overall health. These will be given to you and can be returned in person, via mail or digitally.

- At the measurement visit we will obtain blood, exhaled breath condensate (EBC), saliva and naso/oropharyngeal sampling which will be stored in the NYU-FDNY WTF Biorepository.

Final Study Visit (this visit will occur by phone and will last no more than 15 minutes)

- This visit will occur within 1 year of the Measurement Visit.
- Follow-up questionnaires.

None of the data / specimens generated as part of this study will be added to the FDNY WTC Database or repository.

STORAGE OF DATA AND BIOSPECIMENS FOR FUTURE RESEARCH

With your permission we will draw about 30ml or 2 tbsp of blood and store this blood for future research in the NolanLab NYU-FDNY Repository in Dr. Nolan's laboratory in New Bellevue 16 North Room 20. An 8-12 hour fast will be required of you prior to this draw. Water is permitted during the fasting period. We will also store your study data, EBC, saliva, and naso/oropharyngeal samples if you agree.

Researchers will use the samples to determine the levels of biomarkers associated with WTC dust exposure. We will save the samples in a freezer for future biomarker studies for at least 10 years or until the end of the study. The stored specimen and health information from the FDNY WTC Database / repository will be made available only to researchers directly related to this project. All samples will be coded. "True" genetic testing will not be performed on the samples (testing done in a certified lab to diagnose your predisposition to conditions you don't currently know you have.)

Identifiers will be removed from your identifiable data and specimens. After such removal the data and specimens may be used for future research studies or shared with other researchers and we will not request additional informed consent from you to use these data and specimens as we have noted here.

The results of these future research tests are experimental and will not be released to you.

Please mark your choice below:

☐ **Yes**, I allow my blood and saliva samples to be stored for future use.

☐ **No**, I do not allow my samples to be saved for future research

5. What are the possible risks or discomforts?

A. Risks of Blood Draw: A total of 1 ounce, which is equal to 2 tablespoons or 30 cc, will be taken during the study. Risks include slight pain, bruising, redness, or a small risk of infection at the site of the puncture. Blood will be sampled after an 8-12 hour fast. You may feel dizzy or lightheaded after fasting.

B. Risk of Saliva Sampling: No expected risks.

C. Risks of Fe_{NO} Measurement: Fe_{NO} measurement requires repetitive exhalation and therefore may cause dizziness.

Approved For Period: 4/25/2024 - 4/24/2025

D. **Risks of EBC:** measurement requires repetitive exhalation and therefore may cause dizziness.

E. **Risk of Obtaining Microbiome Samples:** Since these will be obtained using swabs there is a risk of discomfort, gagging and/or abrasions.

F. **Risk of Questionnaires:** You may feel inconvenienced or uncomfortable answering these questions.

G. **Risk to Privacy and Confidentiality:** As private information is collected as part of this study, there is a risk to privacy and confidentiality We will take every precaution to protect your identity and personal information.

H. **Risk of Spirometry (pulmonary function testing):** Requires deep and forceful respiratory efforts. You may experience chest soreness the day following the procedure or light-headedness during the procedure. This risk is mitigated by having you perform the test in the seated rather than standing position.

I. **Clinical Implications:** Showing that biomarkers may predict GERD and/or BE could be useful both in the diagnosis and eventual treatment of WTC associated disease.

J. **Incidental Findings:** If you are found to have treatable conditions (based on your clinical blood tests that include lipids, chemistries, complete blood counts and liver function tests), the PI will contact you by phone or email within 24 hours of these findings, inform you of the results, and direct you to reach out to your primary care physician. If you do not have a primary care physician, we will arrange for you to receive counseling if needed and give you information on how to pursue further care. The incidental findings will not be added to your records in the FDNY WTC Database. If the incidental findings require you to seek follow-up testing or care, you will be financially responsible for these costs.

K. **Risk of Email Communication:** Email communication, despite our best efforts, may be insecure. Under certain circumstances, such as a court subpoena or communications suggesting that a subject or others are at risk of harm by being enrolled in this study, your email communications may be released to others.

L. **Unforeseeable Risk.** In addition to these risks, this research may bring harm in ways that are unknown. These may be a minor inconvenience or may be so severe as to cause death.

6. What if new information becomes available?

During the course of this study, we may find more information that could be important to you. This includes information that might cause you to change your mind about being in the study. We will notify you as soon as possible if such information becomes available.

7. What are the possible benefits of the study?

You will not get any benefit from being in this research study. Others with *WTC*- associated aerodigestive disease may benefit in the future from what we learn in this study.

8. What other choices do I have if I do not participate?

You have the choice not to take part in this study and to receive any necessary medical care outside of this research.

Approved For Period: 4/25/2024 - 4/24/2025

9. Will I be paid for being in this study?

Biospecimens collected for the purposes of this research (even if identifiers are removed) may be used for commercial profit. If your biospecimens are or become commercially profitable, you *will not* share in this commercial profit.
You will be paid $25.00 (at the time of your measurement visit) for your time and effort.

You are required to track all payments made to you by NYU Langone for your participation in any research for this calendar year. You must let us know immediately if/when the total research payments presently equal or is likely to exceed $600.00 total (not including travel reimbursements) for this calendar year. If your total payments (for one or more studies) reach $600.00, please advise *Dr Anna Nolan,* 646-501-6783.

In order to receive payments for your participation in research, you may need to provide your Social Security number. This is because NYU Langone is required to report to the Internal Revenue Service (IRS) any amounts that are paid to research participants that are equal to or greater than $600.00, and you may be taxed on these research payments above $600.00. If you will receive payments in any amount by a check, you will need to provide your Social Security number or Alien Registration number and will be asked to complete an IRS W9. If you do not have either of these numbers or are not willing to complete the IRS, you may be in the study but will not receive any payment.

10. Will I have to pay for anything?

You / your health insurance will not be billed for any study activities, tests, or procedures. The cost of all procedures and tests will be covered by funds received from the CDC/NIOSH Grant Support. Transportation to/from study visits are not provided by the study. Any additional costs accrued for personal phone utilization/cellular data, will not be provided by the study.

11. What happens if I am injured from being in the study

For medical emergencies contact 911. If you think you have been injured as a result of taking part in this research study, tell the principal investigator as soon as possible. The principal investigator’s name and phone number are listed at the top of page 1 of this consent form.

The federal government has a program that may provide compensation to you or your family if you experience serious physical injuries or death. To find out more about this “Countermeasures Injury Compensation Program” go to https://www.hrsa.gov/cicp/about/index.html or call 1-855-266-2427.

We will offer you the care needed to treat injuries directly resulting from taking part in this research. We may bill your insurance company or other third parties, if appropriate, for the costs of the care you get for the injury, but you may also be responsible for some of them.

There are no plans for the NYU School of Medicine or Medical Center to pay you or give you other compensation for the injury. You do not give up your legal rights by signing this form.

12. When is the study over? Can I leave the Study before it ends?

This study is expected to end after all participants have completed all visits, and all information has been collected. This study may also be stopped or your participation ended at any time by your physician, the study sponsor, or the Food and Drug Administration (FDA) without your consent because:

- The principal investigator feels it is necessary for your health or safety. Such an action would not require your consent, but you will be informed if such a decision is made and the reason for this decision.

Approved For Period: 4/25/2024 - 4/24/2025

- You have not followed study instructions.
- The study sponsor, the principal investigator, the Food and Drug Administration (FDA) or other body responsible for monitoring the safety of the study has decided to stop the study.

If you decide to participate, you are free to leave the study at any time. Leaving the study will not interfere with your future care, payment for your health care or your eligibility for health care benefits.

13. How will you protect my confidentiality?

Your medical information is protected health information, or “PHI”, and is protected by federal and state laws, such as the Health Insurance Portability and Accountability Act, or HIPAA. This includes information in your research record as well as information in your medical record at NYU Langone Health. In compliance with NYU Langone Health policies and procedures and with HIPAA, only those individuals with a job purpose can access this information.

Medical information created by this research study may become part of your medical record. We may include your research information in your medical record for several reasons, including for the billing of services provided in connection with the study, to securely document any medical services you receive, and so that other members of the NYU Langone Health community who may treat you have access to important information about your health.

You have a right to access information in your medical record. In some cases, when necessary to protect the integrity of the research, you will not be allowed to see or copy certain information relating to the study while the study is in progress, but you will have the right to see and copy the information once the study is over in accordance with NYU Langone Health policies and applicable law.

Certificate of Confidentiality

To help us further protect your confidentiality, this research is covered by a Certificate of Confidentiality from the National Institutes of Health (NIH). The NIH has issued a Certificate of Confidentiality for this research. This adds special protection for the research information (data, documents, or biospecimens) that may identify you.

Research information protected by this Certificate of Confidentiality cannot be disclosed to anyone else who is not connected with the research, without your consent. With this Certificate of Confidentiality, the researchers may not disclose or use research information that may identify you in any federal, state, or local civil, criminal, administrative, legislative, or other action, suit, or proceeding, or be used as evidence, for example, if there is a court subpoena, without your consent. However, disclosure, without your consent, is still necessary if there is a federal, state, or local law that requires disclosure (such as to report child abuse or communicable diseases).

The Certificate of Confidentiality cannot be used to refuse a request for information from appropriate government agencies responsible for project oversight.
The Certificate of Confidentiality does not prevent you from releasing information about yourself and your involvement in this research, including for your medical treatment. Federal regulations may also allow for the use or sharing of information for other scientific research.

14. HIPAA Authorization

As noted in the Confidentiality section above, federal law requires us, and our affiliated researchers, health care providers, and physician network to protect the privacy of information that identifies you and relates to your past, present, and future physical and mental health conditions. We are asking for your permission

Approved For Period: 4/25/2024 - 4/24/2025

(authorization) to use and share your health information with others in connection with this study- in other words, for purposes of this research, including conducting and overseeing the study.

Your treatment outside of this study, payment for your health care, and your health care benefits will not be affected even if you do not authorize the use and disclosure of your information for this study.

What information may be used or shared with others in connection with this study?
All information in your research record for this study may be used and shared with those individuals listed in this section. Additionally, information in your medical record that the research team believes may be important to the study may be accessed by those listed here. This includes, for example, results from your physical examinations, laboratory tests, procedures, questionnaires, and diaries.

Who may use and share information in connection with this study?
The following individuals may use, share, or receive your information for this research study:

- The research team, including the Principal Investigator, study coordinators, and personnel responsible for the support or oversight of the study
- Governmental agencies responsible for research oversight (e.g., the Food and Drug Administration or FDA).
- The study sponsor: *CDC/NIOSH*
- Health care providers, including your doctors and others who provide services to you in connection with this study, and laboratories or other individuals who analyze your health information in connection with this study.

Your information may be re-disclosed or used for other purposes if the person who receives your information is not required by law to protect the privacy of the information.

What if I do not want to give permission to use and share my information for this study?
Signing this form is voluntary. You do not have to give us permission to use and share your information, but if you do not, you will not be able to participate in this study.

Can I change my mind and withdraw permission to use or share my information?
Yes, you may withdraw or take back your permission to use and share your health information at any time for this research study. If you withdraw your permission, we will not be able to take back information that has already been used or shared with others. To withdraw your permission, send a written notice to the principal investigator for the study noted at the top of page 1 of this form. If you withdraw your permission, you will not be able to stay in this study.

How long may my information be used or shared?
Your permission to use or share your personal health information for this study will never expire unless you withdraw it.

15. The Institutional Review Board (IRB) and how it protects you

The IRB reviews all human research studies – including this study. The IRB follows Federal Government rules and guidelines designed to protect the rights and welfare of the people taking part in the research studies. The IRB also reviews research to make sure the risks for all studies are as small as possible. The NYU IRB Office number is (212) 263-4110. The NYU School of Medicine's IRB is made up of doctors, nurses, scientists, and people from the community.

Approved For Period: 4/25/2024 - 4/24/2025

16. Permission to contact you about future research

With your permission, we might contact you about taking part in future studies. You can decide whether or not you're interested in participating in a particular study when we contact you. You can decline at any time.

☐ I **agree** to be contacted by the study team as described above.

☐ I **do not** agree to be contacted by the study team as described above.

_____ Subject Initials

17. Who can I call with questions, or if I'm concerned about my rights as a research subject?

If you have questions, concerns or complaints regarding your participation in this research study or if you have any questions about your rights as a research subject, you should speak with the Principal Investigator listed on top of the page 1 of this consent form. If a member of the research team cannot be reached or you want to talk to someone other than those working on the study, you may contact the Institutional Review Board (IRB) at (212) 263-4110.

Any questions I had were answered by: ____________________.

I understand that I am entitled to and will be given a copy of this signed release form.

By signing this release form, I give my authorization for the uses and disclosures of my protected health information as described above.

When you sign this form, you are agreeing to take part in this research study as described to you. This means that you have read the consent form, your questions have been answered, and you have decided to volunteer.

Name of Subject (Print)	Signature of Subject	Date

Name of Person Obtaining Consent (Print)	Signature of Person Obtaining Consent	Date

Approved For Period: 4/25/2024 - 4/24/2025
